# Exploring the relationship between employee engagement and counterproductive work behaviour

**DOI:** 10.3389/fpsyg.2024.1434350

**Published:** 2025-01-27

**Authors:** Sphindile Mvuyana, Thokozani Ian Nzimakwe, Reward Utete

**Affiliations:** ^1^Department of Public Management, University of KwaZulu-Natal, Durban, South Africa; ^2^Department of Industrial Psychology and People Management, University of Johannesburg, Johannesburg, South Africa

**Keywords:** employee engagement, induction, probationary periods, promotions policy, organisational norms

## Abstract

**Introduction:**

Despite the cumulative increase in counterproductive work behaviour in various organisations which have ripple effects on general organisational performance, little is known on how employee engagement relates to counterproductive work behaviour. The purpose of the study was to explore the relationship between employee engagement and counterproductive work behaviours of employees at the leading university in South Africa.

**Methods:**

The study employed qualitative approach and structured interviews were used to gather data from the both academic and professional services staff. The collected data from fifteen (15) respondents were analysed using thematic analysis, leading to the identification of key themes and patterns.

**Results:**

Based on the findings, it became evident that there is an inverse relationship between employee engagement and counterproductive work behaviour. The study also revealed lack of an effective probationary period, unequal treatment of academic and professional services staff in terms of retention strategies, and insufficient attention given to employee engagement were among the primary factors contributing to disengagement within the institution.

**Discussion:**

The study concluded that there is a pressing need for change and the implementation of new systems to enhance employee engagement at the organisation. Management should also establish a probationary period for new employees, providing sufficient time for them to acclimate to their roles and allowing line managers and HR to identify and address training needs. Management should enhance communication and feedback channels between employees and management to foster a sense of inclusiveness and ensure that employee concerns are heard and addressed.

## Introduction

Although studies have been conducted on employee engagement and counterproductive work behaviour different sectors, little attention has been given in public sector. Employee engagement is linked to a variety of positive behaviours and outcomes which include job satisfaction, intent to stay, high productivity, job performance, and customer satisfaction. Organisations with engaged employees benefit from increased productivity, higher financial returns, lower turnover, a larger talent pool, higher morale, the production of emotional engagement, high efficiency and loyal customers (Chen et al., 2020). The ability of an organisation to manage employee engagement is inextricably linked to its ability to achieve high performance levels and superior business results through increased productivity. According to Aqil et al. (2023), employee engagement is a result of a combination of satisfaction, motivation, and effectiveness. As a result, job satisfaction, motivation, and effectiveness are three factors that influence employee engagement. The ability of a company to manage employee engagement is directly related to its ability to achieve high performance levels and greater business results.

Undoubtedly, job satisfaction for employees is contingent upon feeling valued and recognised for their contributions. However, for engagement purposes, performance management system fails to reflect the importance of employee opinions, resulting in lack of clarity regarding expectations for both new and existing employees, whether in academic or professional service roles (Huang et al., 2021). Objectives are set prematurely, without first providing proper induction and probationary periods that allow employees to acclimate to their roles. During this crucial period, line managers and human resources department are expected to identify training needs to support the new staff members in meeting their probationary objectives and fostering engagement with their work and the organisation (Hadlington et al., 2021). Furthermore, employee retention exacerbates the engagement deficit, particularly among professional service staff, as more attention appears to be given to academic staff who have opportunities for promotion through the academic promotions policy. Employee engagement in any organisation also creates a comprehensive two-way communication process that breaks down all barriers (Noermijati et al., 2021; Manjoo et al., 2023).

The study sought to identify insights that can inform strategies to cultivate higher levels of positive engagement. Failure to conduct this study poses a significant risk, as low levels of engagement can lead to high employee turnover, compromising the institution’s ability to operate at an optimal level and potentially tarnishing its long-standing reputation (Sypniewska, 2020; Lubbadeh, 2021). Employee engagement plays a pivotal role in three aspects of improvement. Firstly, it serves as an effective strategy to enhance performance and mitigate counterproductive work behaviour (Filipkowski and Derbis, 2020). Researchers universally agree that employee engagement has a positive impact on performance, underscoring the need to review the effectiveness of the current institution’s employee engagement and work behaviour. Despite the cumulative increase in counterproductive work behaviour in South African tertiary institutions which have ripple effects on general organisational performance (Van Niekerk et al., 2017), no study has been conducted to investigate how employee engagement relates to counterproductive work behaviour. Hence, this study to fill this void by exploring relationship between employee engagement relates to counterproductive work behaviour.

The key objectives of this study include:

i) To explore the relationship between employee engagement and counterproductive work behaviour of employees at the institution;
ii) To analyse the effectiveness of the current induction programme of employees at the institution; and
iii) To evaluate the implementation of the current retention strategy of employees at the institution.

## Literature review

### The concept of employee engagement

Employee engagement encompasses the positive attitude that employees hold towards the organisation and its values (Zhao et al., 2022). Furthermore, an engaged employee demonstrates awareness of the business context and collaborates with colleagues to enhance job performance for the organisation’s benefit. Aburub (2020) states that employee engagement involves effectively harnessing the commitment of organisational members to their work roles, where individuals express themselves physically, cognitively, and emotionally during role performances. Building upon the concept introduced by Schaufeli and Bakker (2004), Verčič (2021) defines employee engagement as a positive and fulfilling work-related state of mind characterised by vigour, dedication, and absorption. Employee engagement reflects the level of commitment and involvement an employee exhibits towards their organisation and its values. Consequently, employee engagement is influenced by a complex interplay of interconnected factors (Jabeen and Rahim, 2021). The most frequently reported drivers included confidence and integrity, the nature of the job, the connection between individual performance and corporate performance, opportunities for career growth, pride in the company, relationships with colleagues, employee development, and personal interactions with management.

Several studies have examined the causes and consequences of employee engagement in various contexts. For example, Lartey (2021) investigated employee engagement in the United States of America, aiming to understand the impact of career planning, employee autonomy, and manager recognition on it. Albrecht et al. (2021) focused on the relationship between employee engagement, meaningful work and job resources in Australia. Tao et al. (2022) studied the enhancement of employee engagement via leaders’ motivational language in times of crisis in United States of America. Ababneh (2021) concentrated on the role of employee engagement and personality attributes in Jordan. By focusing on engagement strategies, organisations can enhance various aspects of their effectiveness, including increased productivity, profits, quality, customer satisfaction, employee retention, and adaptability (Engidaw, 2021; De-la-Calle-Durán and Rodríguez-Sánchez, 2021).

Employee engagement is a matter of global concern for leaders and managers, given its recognised role as a critical factor in determining organisational effectiveness, innovation, and competitiveness (Aburub, 2020). Although primarily regarded as a practical consultancy issue, employee engagement has its roots in academic research and has evolved significantly over time. Kahn (1990), one of the early pioneers in theorising work-related engagement, characterised engaged employees as fully connected physically, cognitively, and emotionally with their work roles. Moore and Hanson (2022) define engagement as the process of effectively harnessing organisational members’ personalities to their work roles, where individuals emotionally, physically, and cognitively express themselves during role performances. Kahn (1990) identified meaningfulness, safety, and availability as the primary domains explaining individuals’ engagement in work. Meaningfulness refers to an individual’s self-investment in role performance, enhancing a positive sense of self-fulfilment. Safety pertains to the opportunity for individuals to freely express their true selves without fearing negative consequences to their self-image, career, or status. Availability refers to the possession of emotional, physical, and psychological resources necessary for task completion. Employee engagement, according to Kahn’s multifaceted construct, is influenced by the extent to which individuals invest themselves in their roles, leading to more satisfying and effective performance. Engidaw (2021) states that engagement is a positive and fulfilling work-related state of mind characterized by vigor, dedication, and absorption, which aligns with Kahn’s conceptualization.

### Counterproductive work behaviour

Any employee activity that undermines a business’s goals and interests is referred to as counterproductive work behaviour (CWB) (Chen et al., 2020). Lateness, theft, fraud, sexual harassment, workplace bullying, absenteeism, substance abuse, workplace hostility, and sabotage are just a few examples of counterproductive work behaviours (Hadlington et al., 2021; Ali and Johl, 2020). There is no question that CWBs contradict organisational norms, are harmful to the organisation’s interests, and obstruct the achievement of the organisation’s overall goals. Deviance, antisocial behaviour, unruliness, destructive and hazardous behaviours have all been used to describe CWBs, which have been shown to be pervasive and costly to both organisations and employees’ well-being (Sypniewska, 2020; Chen et al., 2020). Antisocial behaviour, unproductive behaviour, dysfunctional behaviour, and organisational misconduct are all synonyms for CWB (Kundi and Badar, 2021). CWB is also characterised as voluntary behaviour that breaches significant organisational standards and, as a result, endangers the wellbeing of an organisation, its members’, or both.

According to Shen and Lei (2022), counterproductive work behaviour is a discretionary behaviour that disregards the significant norms of the organisation thus threatening the well-being of the organisation or its members, or both. Organisational norms consist of basic moral standards as well as formal and informal organisational policies and procedures. As a result, a relationship between employee engagement and counterproductive work behaviour is likely to exist. Employees are likely to exhibit deviant behaviour in response to negative perceptions of the work situation (Wallace and Coughlan, 2023; De Clercq et al., 2021). It is without a doubt that individuals who are engaged in their jobs, maintain a positive perception of the work (i.e. enthusiastic, satisfied, and interested) while individuals who are not engaged may have a negative perception of their situation at work (i.e., hostility, distressed, irritability). When employees work under unfavourable work environment, they are more likely to engage in CWB (Lipińska-Grobelny, 2021; Mehmood et al., 2022). CWB as a deviant behaviour destructive and harmful to the organisation and its members has many dimensions of acts identified however, for the purposes of this study the researcher has focused on two targets namely the organisation and its employees. Employees’ disruptive actions range from simple activities such as talking back or embarrassing co-workers, leaving work early, or being late, to more serious acts like as sabotage, theft, and so on. CWB is undoubtedly unwelcome and unexpected.

### The role of employee engagement in anticipating CWB

Employee engagement and unproductive work conduct are diametrically opposed. Increasing in one reduce the other. It indicates that as employees become more engaged, they will be less likely to participate in detrimental job conduct (Zhao et al., 2022). As long as employee involvement is strengthened and sustained, the organisation is safe from dangerous and destructive behaviours. On the other side, if the organisation’s personnel are not engaged, it will be vulnerable to CWB. Employees that are engaged have a strong physical and psychological connection that is not easily influenced by CWB elements such as injustice or unfairness (Tong et al., 2020). Employees are more engaged and lot more engaged, not just committed, as a result of the successful approach of keeping them away from CWB. Employee engagement is believed to be one step ahead of commitment, and committed employees are less capable of confronting and coping with CWB causes and avoiding harmful behaviours in any way than engaged employees (Hadlington et al., 2021).

CWB is obviously prevented and should be foreseen by undertaking any measures aimed at improving and maintaining employee engagement predictors and/or drivers. As a result, in order to avoid the CWB, employee engagement should be adequately planned from the outset of the hiring process. Because the organisation’s selection of the incorrect personnel will produce new problems and may even be poisonous in the future. Given the importance of employee engagement, particularly during times of transition, it’s important to understand the responsibilities of employee engagement in anticipating and/or coping with CWB that is perceived to be harmful to the business and its employees. The role of employee engagement can be improved in three ways. Firstly, employee involvement is an excellent method for improving performance and keeping people away from CWB (Shen and Lei, 2022). Employee involvement has a beneficial effect on performance, according to the experts. Employees are safe from intentional wrongdoing or detrimental behaviour, as seen by their improved performance. Excellent performance ensures that the organisation will achieve zero-CWB, and engaged employees are the only ones who can drive and own such excellent performance. Secondly, employee engagement is a powerful tool for improving work and/or business outcomes (Kundi and Badar, 2021; Chen et al., 2020).

Only engaged personnel can achieve and support the finest work and business outcomes that the organisation expects. Making them engaged entails producing the greatest possible results and eliminating flaws. The quality of the working environment provided by poorly engaged personnel includes intentional blunders, poor sound quality, poor working communication, and unexpected working situations (Carpenter et al., 2021). Individual, group, and corporate goals are all likely to be met as a result of the extra struggles and discretionary work put in by engaged employees. As a result, there are no defects, resulting in increased efficiency and effectiveness. Finally, in terms of an organisation’s future prospects, employee involvement is the most important weapon for success (Abdullah et al., 2021). Deviant practices, damaging behaviours, destructive and degraded acts make it difficult for organisations to succeed. Such businesses will not have a bright future until they go out of business. Employee engagement, as a crucial tool for success, motivates all aspects of production, keeps the organisation on course for a bright future, and opens the golden gate to the golden future with golden people.

## Theoretical framework

Considering that the focus of this research is on employee engagement and unproductive work behaviour, two theories have been identified to guide this research. First, the researcher discusses Self-Determination Theory (SDT), which suggests that people are motivated to grow and change by three basic and universal psychological needs. In the Needs-satisfying approach, engagement occurs as a result of a job that is challenging and meaningful, a safe social environment at work and adequate personal resources.

### Self-determination theory (SDT)

The theory of work engagement, Self-Determination Theory (SDT), was formally introduced in the mid-1980s by Deci and Ryan (2012) to examine employee motivational factors. Deci and Ryan developed the Self-Determination Theory, which has been used in professional and academic research that relates to employee engagement. The Self-Determination Theory is concerned with natural or inherent tendencies to behave in healthy and effective ways. The SDT and the essence of work engagement are linked by employee engagement and human behaviours. The ability of an employee to control personal behaviours and goals determines his or her level of engagement.

Disengagement and personal engagement are linked to the SDT in the sense that an employee’s behavioural state is a key motivator for demonstrating behaviour at both the professional and personal levels. An organisation’s productivity is influenced by its employees’ level of engagement. An employee’s motivation is related to his or her job satisfaction. Employee motivation is also influenced by their emotional state. Employees become disengaged and defensive when they begin to withdraw and conceal their identities, ideas, and feelings, which has a negative impact on work performance (Engidaw, 2021). Business leaders’ implementation of employee engagement strategies results in higher levels of employee engagement, customer satisfaction, productivity, and profit (Albrecht et al., 2021), and lower levels of employee accidents and turnovers (Linggiallo et al., 2021). Business leaders use SDT to help employees develop positive attitudes toward their company (Kaur and Mittal, 2020). The Self-Determination Theory is therefore relevant to the study because when employees are engaged, their number one objective is to contribute to the company’s success; they also understand that it is not only about the benefits or bonuses but also about being part of a successful business. Employee engagement cannot be overstated; employee engagement strategies have been shown to reduce staff turnover, increase productivity, and efficiency in the workplace.

### The needs-satisfying approach

Employees become engaged, according to Kahn (1990), when three psychological conditions or needs are met: meaningfulness (the feeling of receiving a return on one’s self-investments in role performance), psychological safety (the feeling of being able to show and employ one’s self without fear of negative consequences), and availability (i.e., the belief of having the physical and mental resources to engage the self at work). The nature of the job, including the task and role qualities, has an impact on its meaning. The social environment, which includes interpersonal connections, group dynamics, managerial style, and societal norms, has the greatest impact on psychological safety (Engidaw, 2021). Finally, availability is determined by people’s personal resources, such as physical energy, that they can contribute to their role performance. As expected, meaningfulness, as well as, to a lesser extent, safety and availability, were positively linked with involvement. They also discovered that job enrichment and role fit were positively connected to meaningfulness, while rewarding co-worker and supportive supervisor relationships were positively related to safety, and personal resources were positively related to availability, in agreement with Kahn’s theory.

## Methods

### Research approach and design

This study adopted a qualitative approach and exploratory research design. Qualitative research is concerned with developing explanations of social phenomena. It aims to help us to understand the social world we live in and why things are the way they are (Lanka et al., 2020). This study is qualitative in nature because it is aimed at getting a deeper understanding and views of both academic and professional services staff regarding employee engagement at the institution under the study. Qualitative research is appropriate for this study because it is based on the exploratory nature of the phenomenon being examined, with the objective of coming up with a rich, descriptive, and presentable picture of the study. Qualitative research is essentially embedded in the relationship between the phenomenon and how people conceive or interpret it to be in their experience (Tomaszewski et al., 2020). For the purposes of this study, only qualitative research method was adopted.

### Respondents

For this study, the target population selected ranged from academic staff in the five campuses and the professional services staff in different positions based on hierarchy levels with some focus on HR leadership positions. The target population was academic staff and professional services from a public tertiary institution in South Africa. The target population was 4,000. According to Creswell and Creswell (2018), under qualitative research study a sample between 10 and 50 participants may be considered. Dzwigol (2022) defines a sample as a selected group of the entire population that you wish to study. In this study, the sample comprised of 20 employees broken down into 9 academics and 11 professional services staff. However, the participants who voluntarily participated in this study were fifteen (15). Owing to the limitation of accessibility, convenience sampling technique under the ambit of non-probability sampling was used to select the sample. For a well-functioning higher education institution, it is important to have a well-functioning administration. This cannot be achieved without the professional services staff as academics have different key performance indicators and objectives. It is therefore imperative for the study to focus on both sectors to provide insight as in most cases, professional services staff hold more corporate knowledge required to ensure that the organisation performs to its optimal level. While the focus of Academic staff may be on students, the professional services staff are the main drivers of employee engagement. It is also important to note that Academics and Professional Services staff may be motivated and engaged by different factors at institution and therefore it is important for both sides to be heard.

### Data collection methods

The main research method of this study, has one principal methods of collecting data which is-interviews (Zhang et al., 2023). This study conducted semi-structured interviews with 15 participants from Professional Services and academic staff. The interview method of data collection entails presenting oral-verbal stimuli and responding in terms of oral-verbal responses. This method can be used for personal interviews as well as, if possible, telephone interviews (Pandey and Pandey, 2021). The interviews were structured as such that professional services staff were interviewed separately from the academic staff. The data collected from the interviews was used as part of the data analysis for the study.

### Data analysis

Data was manually analysed by following qualitative data analysis systematic steps. Thematic analysis was employed to analyse the data is to get a clear sense of the relevant data that have been collected from participants. Thematic analysis organises and analyse empirical data gathered from interviews. The gathered data was translated, transcribed and then coded. According to Byrne (2021), the six-step phase was followed as a method for conducting thematic analysis. The initial phase was preparation the data for analysis. In this study, the voice recorder was used to record all the responses. All audio-recorded interviews were compiled into one location. The researchers ensured that the recordings were saved safely. Data transcription was the second phase. In this phase, the researchers utilised the service of professional transcriber to transcribe all the recordings from the respondents. The researchers listened to the recordings three times to check if the transcriptions were well captured. All the transcriptions were later saved safely in one file. In addition, the notes that were jotted during were also put together with transcriptions. Familiarising with data became the third phase process of analysing the data. At this phase, the researcher thoroughly read the transcriptions in order to derive meaning from them. Memoing the data formed part of the fourth phase. After reading the transcribed data for further analysis, the researcher jotted down the key emerging data interpretations. Data coding was the fifth phase of the analysis in this study. The study utilised Microsoft Excel to code and analyse transcribed data from the respondents. The data was analysed manually using Microsoft Excel. Moving categories to themes was the sixth phase of thematic analysis. The researchers developed categories from codes and then themes were produced from categories. Based on the transcribed data, the researchers manually extract main themes and sub-themes using Microsoft Excel in line with the research objectives. In this study, researchers developed a comprehensive narrative that explains each theme in detail. The phases assisted the researchers to derive insights and sound interpretations on how each theme contributes to the understanding of the relationship between employee engagement and counterproductive work behaviours.

### Ethical considerations

Crabtree and Miller (2023) explain that ethics is a moral or professional code of conduct that sets the standard for the attitudes and behaviour of the researcher/s and participants. Certain ethical concerns, such as plagiarism and honesty in reporting of results, arise in all research but additional issues arise when the researcher involves human subjects which in this study humans will be subjected to an interview data collection method, it is therefore imperative to note that, the privacy during the interview sessions and confidentiality of all participants who had agreed to participate was prioritised at all times. The gatekeepers’ letter to conduct the study, was sought and issued by the Registrar’s office at the institution. Ethical clearance application was then lodged with the Research Office and the ethical clearance letter was subsequently approved and issued by the University Research Ethics Committee.

## Results and discussion

### Profile of the respondents

The demographic characteristics of the respondents featured in the study include years in the position, ranking and ages of respondents. Of sixteen (15) participants interviewed nine (9) were females and six (6) males. Table 1 clearly illustrates the nature of the participants that the researcher interacted with when conducting the research interviews. The letter P above represents ‘Participant’. So, P1, for instance, stands for Participant 1.

**Table 1:** Profile of the respondents.

Participant	Gender	Age	Race	Rank	Classification	Years in service
P1	F	60	African	Director	Professional Services	19
P2	F	40	African	Administrator	Professional Services	5
P3	M	40	African	Lecturer	Academic	10
P4	M	63	Indian	Director	Professional Services	2
P5	F	46	White	Senior Lecturer	Academic	15
P6	F	39	African	Director	Professional Services	9
P7	M	63	African	Professor	Academic	15
P8	M	48	African	Lecturer	Academic	4
P9	M	36	African	Administrator	Professional Services	7
P10	F	38	African	Professor	Academic	7
P11	F	38	African	Manager	Professional Services	3
P12	F	39	White	Lecturer	Academic	6
P13	F	50	African	Senior Administration	Professional Services	13
P14	F	45	Mixed race	Senior Administration	Professional Services	10
P15	M	30	African	Junior Administration	Professional Services	2

Source: Authors’ analysis.

The study indicates that the ages of the participants were between 30 and 63 years. In terms of the rank, nine (9) participants were from professional services and six (6) were from the academic space. The researcher conducted the interviews using an interview schedule, which comprised of 8 standard open-ended questions. In addition, the participants were given an opportunity to provide any final comments at the end of their one-on-one interviews. The researcher was thus able to acquire additional information associated with the research topic. The interview questions were aimed at responding to the research objectives, which were as follows: (i) explore the relationship between employee engagement and counterproductive work behaviour of employees at the institution; (ii) analyse the effectiveness of the current induction programme of employees at the institution; (iii) evaluate the implementation of the current retention strategy of employees at the institution.

### Analysis of data

The primary role of data analysis in research is to obtain a comprehensive understanding of the relevant data collected from participants, documents, and other sources. This is essential because not all the information gathered is included in the study’s findings; only the most pertinent data are utilized (Verma et al., 2024). In the context of this particular study, the data collection method employed was interviews, as it facilitates effective expression by the interviewees. This approach enabled the interviewer to gain a deeper comprehension and exploration of the opinions, personalities, experiences, and phenomena of the research subjects. Open-ended interview questions were utilised to gather detailed information. To organise and analyse the empirical data acquired from these interviews, a thematic analysis process was employed. Thematic analysis is a qualitative data analysis method that involves systematically searching through a dataset to identify, analyse, and report recurring patterns (Nayak and Singh, 2021). In this study, themes emerged from the collected data. This is because not all the information gathered is presented in the findings of the study, that is only the most relevant data are used (Jahja et al., 2021). Some responses from the respondents were quoted verbatim to reinforce and augment the presentation without altering the original statements from the participants. Dissimilar information and contradictory information was synthesised into diverse themes.

#### Objective 1: to explore the relationship between employee engagement and counterproductive work behaviour of employees at the institution

##### Theme one: employee engagement and work behaviour

The majority of the respondents indicated a consensus regarding the existence of the relationship between employee engagement and work behaviour. The respondents P1; P3; P4; P5; P6; P8; P10; P11; P12 and P13 agreed that there is a positive association between employee engagement and work behaviour. However, respondents P2; P7; P14 and P15 could not confirm the relationship between the two variables. This entails that an engaged employee creates some willingness and emotional attachment therefore inclined to maintain the good image of the organisation. Most respondents indicated that the aspects of employee engagement are closely related to aspects of innovative work behaviour. Hence, the behaviour of engaged employee usually aligns with objectives of the organisation. Participant 11:

*From my understanding, engaged employees display positive and productive work behaviour including discretionary behaviour and these may be indicative of organisational citizenship. Disengaged employees on the other end may exhibit counterproductive work behaviour (CWB) which may include but not limited to tardiness, theft, fraud, sexual harassment, workplace bullying, absenteeism, substance abuse, workplace aggression, sabotage, or inappropriate use of any of the company resources. Based on my understanding I would therefore say; interrogating indicators of these behaviours may provide a better-informed view/picture of this relationship within the institution. It should be noted however, that although this may provide an overview of the CWB in UKZN, it may not be entirely accurate as covert CWBs may be difficult to measure until it’s late and snowballs into a huge corporate scandal tarnishing the organisational image. I do think that the institution experiences all the relevant challenges faced by the Higher Education sector but employees manage this within acceptable levels.*

###### Sub-theme one: strong teamwork and participation

The bulk of the participants (P3; P4; P5; P6; P7; P8; P9; P10; P11; P12; and P15) acknowledged the existence of robust teamwork within the institution, whether limited to their respective departments or extending to the University as a whole. Nevertheless, participants P1; P2; P13; and P15 indicated non-existence of teamwork as they work in silos and also stated that the academic members are seen as more important than support staff members. From the responses, it can be concluded that, indeed, there is existence of strong teamwork and cordial participation. They also concurred that this sense of teamwork is instigated by top management. P3:

*I can say yes, at the school level there is some aspect of teamwork maybe not 100% but the level of teamwork has really improved over the years. We have team building exercises and retreats which really do keep us engaged as a school. In terms of the college of Humanities at large, I can say the DVC is trying his best to ensure teamwork for an example, there is even a WhatsApp group for the leaders but also must point out that not all departments work well together, so improvement is needed.*

###### Sub-theme two: effective communication

Majority of the participants (P1; P2; P3; P5; P7; P9; P11; P12; P13; and P15) indicated that communication within the organisation is ineffective. The participants stated that institution relies on notice boards which most people do not see or read. In some instances, the participants indicated the influx of emails and notices which confuses them. One of the notable ineffective communications is failure to communicate about the stoppage of performance bonuses to the employees. However, some participants (P4; P6; P8; P10; and P14) confirmed that communication in the organisation was effective.

#### Objective 2: analyse the effectiveness of the current induction programme of employees at the institution

##### Theme two: induction programme of employees

Among the 15 participants involved in the study, 11 of them (P2; P3; P4; P5; P6; P7; P8; P9; P12; P13; and P14) expressed dissatisfaction with the effectiveness of the induction programme. However, these participants acknowledged the critical role of induction in facilitating the seamless integration of new employees into their respective roles and the organisation as a whole. The majority of the respondents indicated that departmental induction carried out by the line manager is more crucial than the general induction conducted by the human resource personnel team. The respondents stated that the general induction usually takes too long to happen, normally after three (3) which makes it useless to the new recruits. P12:

*Sadly, I am unaware of any employees having taken part in any induction programmes. In the past two years I have had three new employees come under my line management, and as far as I’m aware, none have been invited for induction. Additionally, when I was taken on in a full-time capacity by the University in 2016, I was not offered any opportunity to engage in an induction programme. I believed it did not exist until I heard in mentioned by an HR rep in a School Board meeting. There is also, as far as I’m aware, any induction for staff who take on leadership positions to help acquaint them with their job description or the expectations that the University has for them in a line manager position.*

###### Sub-theme: training programmes

An overwhelming number of participants (P2; P3; P4; P5; P6; P8; P9; P10; P11; P12; P13 and P15) agreed that the institution does provide a wide range of training. However, some participants P1; P7; and P14 indicated that scheduling training sessions poses a challenge as they often overlap with their work responsibilities, which ultimately defeats the purpose as they are unable to attend. In addition, majority of the respondents indicated that their organisation is adopting the new systems late which demotivates them as they not seen as competitive in the academic space. P5:

*Yes, UKZN is very good at arranging trainings when new systems are introduced. However, the timing is always off as it usually clashes with the teaching timetables, and you find that majority of lecturers aren’t able to attend.*

#### Objective 3: evaluate the implementation of the current retention strategy of employees at the institution

##### Theme three: retention strategy of employees

Most participants indicated that the organisation’s current retention strategy is ineffective, as it primarily focuses on retaining employees only after they have submitted their resignation. Participants P2; P3; P4; P5; P6; P7; P8; P9; P10; P14; and P15 stated that existing retention strategy is ineffective and some participants were not even aware of the existence of a retention strategy or policy, as they had witnessed the departure of numerous talented individuals from the institution. Most participants confirmed that there is high labour turnover as employees leave for higher pay and their organisation is not willing to counteroffer. Some participants indicated that there is work overload and employees rarely get promoted. However, participants P1; P11; P12; and P13 view the current retention strategy is effective. Based on these responses, it can be concluded that the retention strategy employed by organisation is far below their expectation, hence ineffective. P8:

*I cannot say much about the retention strategy as I believe that it is not well communicated. I know nothing at all about this strategy but I do believe that UKZN needs to consult with their employees to find out what makes them happy and what can be done to improve the work environment.*

###### Sub-theme one: recognition for good performance

The majority of participants feel valued by their line managers, they are recognized for their performance to some extent although a lot of improvement is also needed in terms of development and growth opportunities in their roles. The participants P1; P3; P4; P5; P6; P7; P8; P10; P11; P13; P14; and P15 felt they are given adequate recognition for their well-performed work. However, participants P2; P9; and P12 felt not recognised and cited that the most demotivating factor is that performance management system is no longer linked to a pay incentive. The overall view deduced from the responses is that the employees are being recognised in the organised. P1:

*Retentions strategy is effective is particularly in the academic space, I have experienced it in academia where we retain people when they are offered jobs elsewhere by counteroffering and this is how we try to retain the talent. Retention is for high performers within the university when they leave. Other strategies of retention in the way of development and getting people to be excited about work depends largely on how line managers interact with their people. The retention strategy exists and for me it is a good strategy and if implemented well, it will retain people at UKZN. But the other aspect of how people remain engaged in their space, is dependent on their experiences within their own departments. Our core business is academics and we always have a shortage hence the focus is more on the academic space. And for professional services in terms of retaining, the only strategy that works for them is in terms of development, the training that is offered, improvement of their qualifications through studying. They can apply for higher position jobs and also job rotation within the university. For academics if it more about the scarcity of the skill and the competition between the universities for the same skill.*

## Discussion

The findings of the study indicated that there is positive relationship between employee engagement and work behaviour. The results of this study are in alignment with the research conducted by Chen et al. (2020) which found that work engagement decreased counterproductive work behaviour. In addition, the finding of the present study is alignment with the self-determination theory (SDT). The results of this are in congruent with a study carried out Aqil et al. (2023) who found that work engagement had a negative and significant impact on counterproductive work behaviour. Similarly, Lubbadeh (2021) carried a study on work behaviour and found the significant positive relationship between job burnout and counterproductive work behaviour. Notably, workplace culture emerges as a critical factor in determining the level of employee engagement (Lockwood, 2007; Glen, 2006). According to their findings, organisations that are regarded as “employers of choice” tend to foster higher levels of employee engagement. Such organisations prioritise the creation of workplace environments that cultivate feelings of respect and value among employees. Additionally, they establish strong connections between individuals and the organisation, motivating employees to exert their utmost efforts in contributing to its success (Sypniewska, 2020).

The results of the study revealed dissatisfaction of employees about the induction programme. The employees acknowledged the critical role of induction in facilitating the seamless integration of new employees into their respective roles and the organisation as a whole. A successful induction process not only fosters a sense of value and belonging among new hires but also cultivates a familial atmosphere within the organisation. The findings of this study are in tandem with Rabten et al. (2024) found that induction training has a positive impact on the adaptation to the new work environment and individual work performance. In addition, the results of this study concurs with Khanam et al. (2020) who found that induction training programme, and delivery system all have a beneficial impact on employee performance. For instance, Engidaw (2021) posits that organisations considered “employers of choice” exhibit higher levels of employee engagement. Such organisations prioritise creating a workplace environment that upholds respect and appreciation for employees, fostering a strong connection between individuals and the organisation. Consequently, employees are motivated to exert maximum effort in contributing to the organisation’s success.

The current study established that organisation’s current retention strategy is ineffective, as it primarily focuses on retaining employees only after they have submitted their resignation. However, some employees were not even aware of the existence of a retention strategy or policy, as they had witnessed the departure of numerous talented individuals from the institution. This viewpoint is also supported by relevant literature. This findings reinforces a study of Xiong and Wen (2020) who found that turnover intention and work engagement were significantly correlated with organisational citizenship behaviour and counterproductive work behaviour. These results echo the work of De Clercq et al. (2021) who found that unfair organisational treatment leads to enhanced counterproductive work behaviour. Similarly, Mehta et al. (2014) and Ekhsan et al. (2022) found that employee retention positively impacts an organisation’s profitability and enhances its market image and reputation.

The findings of this study indicated that their institution provides them with a wide range of training. However, scheduling training sessions poses a challenge as they often overlap with their work responsibilities, which ultimately defeats the purpose as they are unable to attend. The significance of staff training is supported by existing literature. As stated by Ngema et al. (2022), investing in individuals through training and development enables them to acquire new skills, thereby enhancing their competency levels and facilitating career advancement. Moreover, employees derive substantial benefits from training programs that contribute to their personal and professional growth. In addition, the findings of the study indicated the existence of robust teamwork within the institution, whether limited to their respective departments or extending to the institution as a whole. The results also revealed that the sense of teamwork is instigated by top management. This viewpoint finds support in the works of several authors. Notably, Kaur and Mittal (2020) emphasise the influential role of leaders in promoting employee engagement. These scholars assert that leaders can effectively motivate and inspire employees by exemplifying explicit values and nurturing a positive and harmonious teamwork environment.

## Implications of the study

The results of this study expanded self-determined theory by providing a better understanding of positive relationship between employee engagement and work behaviours. The findings of this study confirm and validate the assumption of self-determined theory that there is a close link between employee engagement and work behaviour. Poor employee engagement promotes counterproductive work behaviours. The absence of a probation period further contributes to disengagement. The probationary period plays a vital role in allowing new employees to familiarize themselves with their roles. During this phase, line managers and human resources should identify training needs to support the new staff members in meeting the probationary objectives, thereby fostering engagement with their work and the organisation.

Moreover, the lack of an effective employee retention strategy, particularly one that applies equally to both Academic and Professional Services staff, exacerbates the issue of disengagement within the university. The management should implement a comprehensive retention strategy that encompasses both Academic and Professional Services staff, addressing the unique needs and concerns of each group. Management should also establish a probationary period for new employees, providing sufficient time for them to acclimate to their roles and allowing line managers and HR to identify and address training needs. Management should enhance communication and feedback channels between employees and management to foster a sense of inclusiveness and ensure that employee concerns are heard and addressed. Management should develop and implement an employee engagement policy that demonstrates the university’s commitment to fostering a positive and engaging work environment. Based on the responses obtained during the data collection phase, it becomes evident that the management must promptly develop an employee engagement policy to enhance the level of engagement within the institution. This policy should be comprehensive and applicable to all employees across all hierarchical levels, ensuring equitable treatment. Implementing such a policy would signify the university’s commitment to fostering employee engagement and promoting a positive work environment.

To facilitate effective implementation, the university should offer training, conduct team-building sessions, and provide adequate resources to line managers responsible for overseeing their teams’ engagement. These initiatives will enable managers to effectively nurture engagement among their team members while aligning individual objectives with those of the organisation. Additionally, the actions taken to promote employee engagement should be derived from employee engagement surveys, which serve as valuable tools for gathering feedback. The survey responses should be regularly published, ensuring transparency and providing all employees with insights into the organisation’s engagement efforts. The policy should explicitly state that the effectiveness of employee engagement initiatives will undergo yearly reviews, enabling the university to assess progress and make necessary adjustments as required. By implementing an employee engagement policy, incorporating appropriate training and resources, and regularly evaluating engagement efforts, the management can create a supportive work environment that fosters high levels of engagement and contributes to the well-being and success of its employees.

The induction of a new employee concludes the recruitment and selection process and marks the beginning of the employee’s career. As stated in the problem statement, the induction programme only takes place after 3 months when a new employee has joined, this defeats the purpose of induction which is to; familiarise new employees with all the aspects of the company and the new job. At the end of the induction process it is expected of the new employee to know all the departments/sites, their functionalities as well as general information surrounding the respective operations. On this basis, it is therefore crucial for management to relook at their current induction programme and ensure that it happens timeously and not after 3 months when the person has already started working.

Based on the research conducted in this study, majority of the respondents felt that the current retention strategy is not effective as the organisation only retains staff when they have expressed their intention to exist the company. Some participants also did not even know there is a retention strategy or policy in place as they have witnessed a lot of talented people exit the institution. Based on the responses, it is therefore recommended for the institution to revisit their policies and ensure that they are well communicated and easily accessible to all staff. Engaged employees are the foundation of any organisation. Management should conduct employee engagement surveys at regular intervals to better understand employee engagement levels. These surveys will not only collect employee responses, but will also provide a first-hand source of data that will lead to actionable insights. It is important for management to understand the purpose of conducting surveys as they are not only created for employees to have a voice and a platform to give honest feedback to the organisation/manager/supervisor about the things that are genuinely not going right, but they are also a good tool to measure employee engagement by getting to the root cause of employee dissatisfaction which will assist the management to direct their energy towards improving how they do things and in turn lead to organisational growth.

To ensure the effective drive of employee engagement initiatives, it would be beneficial for the management to consider establishing the role of an Employee Engagement Manager within its HR department. This designated position would assume the sole responsibility of overseeing and coordinating employee engagement activities throughout the organisation. The primary role of the Employee Engagement Manager would involve holding both line managers and employees accountable for their involvement in employee engagement initiatives. By assuming this responsibility, the manager would play a pivotal role in promoting and facilitating engagement efforts across the institution. One of the key tasks of the Employee Engagement Manager would be to assist in the selection and implementation of appropriate employee engagement software. This would involve researching and identifying software solutions that align with the specific needs and objectives of the organisation. Additionally, the manager would be responsible for educating managers and employees on the effective utilization of the selected software, ensuring its optimal use to drive engagement. Moreover, the Employee Engagement Manager would act as the central point of contact for any engagement-related issues within the organisation. They would serve as a resource and advisor, providing guidance and support to staff members at all levels. By assuming this role, the manager would foster a culture of accountability and ensure that engagement efforts are consistently prioritised and addressed. The above recommendations will go a long way towards assisting the university in addressing the challenges of employee engagement. These ideas of engagement encompass both individual wellbeing and organisational level success. This is because engaged employees are: highly energized and resilient professionals; putting their heart and soul into their job; exhibiting strong work involvement; and experiencing feelings of significance, passion, and excitement.

## Conclusion

The study established the existence of negative relationship between employee engagement and counterproductive work behaviour at the organisation. The results of this study expanded self-determined theory by providing a better understanding of positive relationship between employee engagement and work behaviours. The findings of this study confirm and validates the assumption of self-determined theory that there is a close link between employee engagement and work behaviour. Poor employee engagement promotes counterwork behaviours. In analysing the employee engagement, the study specifically analyses the effectiveness of the current induction programme and the implementation of the retention strategy. The study found lack of an effective induction, probationary period, unequal treatment of academic and professional services staff in terms of retention strategies, and insufficient attention given to employee engagement as among the primary factors contributing to disengagement within the institution. Based on the analysis and findings, it became evident that there is a pressing need for change and the implementation of new systems to enhance employee engagement at the organisation. This may pave way for positive change and fostering a culture of employee engagement that benefits both the university and its workforce. The study extends literature of employee engagement by incorporating the counterproductive work behaviour. The current study went beyond the limitations of prior studies which overlooked the influence of induction and retention strategy. By incorporating induction and retention strategy the study provides new insights in the realm of employee engagement. The study found that sound retention strategy and induction is likely to increase employee engagement.

### Limitations and future research

The study has some limitations that must be overcome in future research. The study employed a cross-sectional study and can be premature to draw clear conclusions on some aspects of employee engagements. Longitudinal study could be recommended for future studies. In addition, the qualitative research does not sufficiently confirm causal links between employee engagement and counterproductive work behaviour, hence future studies may consider quantitative research approach. Furthermore, the data was gathered from only one university hence the results may not be generalised to other universities. For this reason, future studies should consider inclusion of other universities to increase the universality of the conclusions.

## Data Availability

The raw data supporting the conclusions of this article will be made available by the authors, without undue reservation.
